# Enhanced Biofilm Formation by Ferrous and Ferric Iron Through Oxidative Stress in *Campylobacter jejuni*

**DOI:** 10.3389/fmicb.2018.01204

**Published:** 2018-06-06

**Authors:** Euna Oh, Katelyn J. Andrews, Byeonghwa Jeon

**Affiliations:** School of Public Health, University of Alberta, Edmonton, AB, Canada

**Keywords:** *Campylobacter*, biofilms, oxidative stress, iron, survival mechanisms

## Abstract

*Campylobacter* is a leading foodborne pathogen worldwide. Biofilm formation is an important survival mechanism that sustains the viability of *Campylobacter* under harsh stress conditions. Iron affects biofilm formation in some other bacteria; however, the effect of iron on biofilm formation has not been investigated in *Campylobacter*. In this study, we discovered that ferrous (Fe^2+^) and ferric (Fe^3+^) iron stimulated biofilm formation in *Campylobacter jejuni*. The sequestration of iron with an iron chelator prevented the iron-mediated biofilm stimulation. The level of total reactive oxygen species (ROS) in biofilms was increased by iron. However, the supplementation with an antioxidant prevented the total ROS level from being increased in biofilms by iron and also inhibited iron-mediated biofilm stimulation in *C. jejuni*. This suggests that iron promotes biofilm formation through oxidative stress. Based on the results of fluorescence microscopic analysis, Fe^2+^ and Fe^3+^ enhanced both microcolony formation and biofilm maturation. The levels of extracellular DNA and polysaccharides in biofilms were increased by iron supplementation. The effect of iron on biofilm formation was also investigated with 70 *C. jejuni* isolates from raw chicken. Regardless of the inherent levels of biofilm formation, iron stimulated biofilm formation in all tested strains; however, there were strain variations in iron concentrations affecting biofilm formation. The biofilm formation of 92.9% (65 of 70) strains was enhanced by either 40 μM Fe^2+^ or 20 μM Fe^3+^ or both (the iron concentrations that enhanced biofilm formation in *C. jejuni* NCTC 11168), whereas different iron concentrations were required to promote biofilms in the rest of the strains. The findings in this study showed that Fe^2+^ and Fe^3+^ contributed to the stimulation of biofilm formation in *C. jejuni* through oxidative stress.

## Introduction

*Campylobacter* is a leading bacterial cause of gastroenteritis and is responsible for approximately 166 million diarrheal cases and 37,600 deaths worldwide per year (Kirk et al., 2015). In addition to gastrointestinal infections, in some cases, *Campylobacter jejuni* may result in the development of Guillain–Barré syndrome (GBS), an acute flaccid paralysis (Willison et al., 2016). Although *C. jejuni* is isolated from a wide range of domestic, companion, and wild animals (Huang et al., 2015), poultry is considered as the most important reservoir for foodborne transmission of *C. jejuni* to humans (Hermans et al., 2012). Compared to other foodborne pathogens, such as *Salmonella* and pathogenic *Escherichia coli, C. jejuni* is physiologically unique (e.g., microaerophilic and asaccharolytic) and fastidious to culture (Silva et al., 2011). Thus, specific culture conditions are required for the growth of *C. jejuni*. For example, low oxygen concentrations (e.g., 5% O_2_) and high growth temperatures (e.g., 37∼42°C) are needed for the optimal growth of *C. jejuni* (Davis and DiRita, 2008). As a capnophile, additionally, *C. jejuni* requires CO_2_, and carbonic anhydrase that is encoded by *canB* contributes to *C. jejuni* growth under low (such as 1%) CO_2_ conditions (Al-Haideri et al., 2016).

A biofilm is microbial communities that are encased in a matrix of self-produced extracellular polymeric substance (EPS), including extracellular DNA (eDNA), polysaccharides, and proteins (Flemming et al., 2016). *C. jejuni* is capable of forming biofilms on various abiotic surfaces and frequently isolated from environmental samples (Kemp et al., 2005; Jokinen et al., 2011). Particularly, biofilm formation is deemed as an important survival mechanism in *C. jejuni* (Buswell et al., 1998; Murphy et al., 2006). As bacteria are usually found in biofilms in natural settings (Branda et al., 2005), *Campylobacter* is also found in biofilms on the surface of river rock and wood in the environment (Maal-Bared et al., 2012).

Several environmental factors affecting biofilm formation in *C. jejuni* have been reported. The biofilm formation of this microaerophilic bacterium is enhanced under aerobic conditions (Reuter et al., 2010; Turonova et al., 2015). Oxygen-rich conditions enhance the expression of membrane proteins, such as Peb4 and CadF, which are involved in the adhesion of *C. jejuni* to abiotic surfaces (Asakura et al., 2007; Sulaeman et al., 2012). Increased oxidative stress under aerobic conditions is associated with biofilm stimulation in *C. jejuni* (Oh et al., 2016). In addition, biofilm formation is also affected by nutritional factors. For instance, nutrient-rich culture media and high salt concentrations reduce biofilm formation in *C. jejuni* (Reeser et al., 2007). Iron is an essential nutrient required for all organisms (Chandrangsu et al., 2017) and is associated with biofilm formation in some bacteria, such as *Streptococcus mutans* (Berlutti et al., 2004). *Staphylococcus aureus* (Lin et al., 2012), and *Pseudomonas aeruginosa* (Banin et al., 2005). Since iron affects various biological processes in *C. jejuni*, such as gene expression regulation (e.g., Fur regulon) and protein glycosylation (e.g., *pglA, pglC*, and *pglH*) (Palyada et al., 2004), we hypothesized that iron may be involved in biofilm formation in *C. jejuni*. To prove this hypothesis, in this study, we investigated the effect of ferrous (Fe^2+^) and ferric (Fe^3+^) iron on biofilm formation in *C. jejuni* NCTC 11168 and 70 *C. jejuni* strains isolated from raw chicken.

## Results

### Stimulation of Biofilm Formation by Iron

**FIGURE 1:**
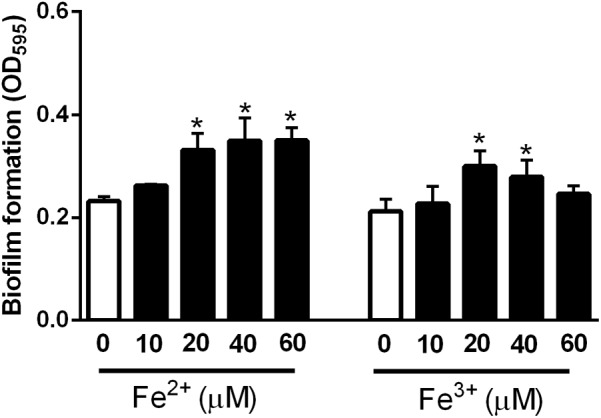
Stimulation of biofilm formation by iron in *C. jejuni* NCTC 11168. The data show the means and standard deviations of three samples in a representative experiment. The experiments were repeated three times, and similar results were obtained in all repeated experiments. The statistical analysis was performed with Student’s *t*-test in comparison with the non-treated sample. ^∗^*P* < 0.05.

To examine the effect of iron on biofilm formation, biofilm assays were performed with minimal essential medium alpha (MEMα), which does not contain iron, with/without iron supplementation. Interestingly, biofilm formation in *C. jejuni* was significantly enhanced by iron (**Figure 1**). Although both Fe^2+^ and Fe^3+^ affected biofilm formation in *C. jejuni*, Fe^2+^ and Fe^3+^ stimulated biofilm formation at different concentration ranges (**Figure 1**). Although the averages of bacterial counts in biofilms were slightly reduced at iron concentrations ≥20 μM, the reduction was not statistically significant, and the viability of *C. jejuni* in biofilms was not altered at the iron concentrations tested in the study (**Supplementary Figure S1A**). These results showed that iron, both Fe^2+^ and Fe^3+^, enhanced biofilm formation in *C. jejuni*.

### Inhibition of Iron-Mediated Biofilm Promotion by a Chelator and an Antioxidant

**FIGURE 2:**
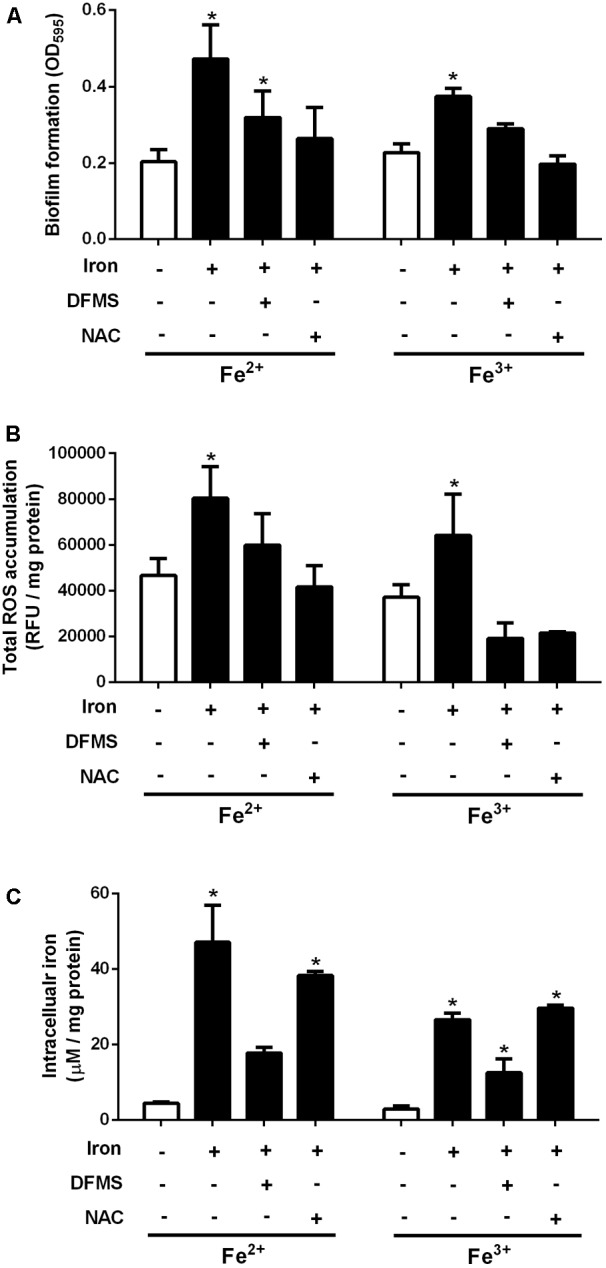
Effects of iron, a chelator, and an antioxidant on the levels of biofilm formation **(A)**, ROS production **(B)**, and intracellular iron **(C)** in *C. jejuni* NCTC 11168. The concentrations of Fe^2+^ and Fe^3+^ were 40 μM and 20 μM, respectively, which were determined based on the results of the biofilm assay (**Figure 1**). Twenty micromolar DFMS and 1 μM N-acetylcysteine (NAC) were used as an iron chelator and an antioxidant, respectively. The results are the means and standard deviations of three samples in a representative experiment. The experiments were repeated three times, and similar results were observed in all repeated experiments. The statistical analysis was performed with Student’s *t*-test compared to the non-treated sample. ^∗^*P* < 0.05.

To confirm the effect of iron on biofilm stimulation, biofilm assays were performed in the supplementation with an iron chelator. The treatment of biofilms with an iron chelator significantly inhibited the iron-mediated enhancement of biofilm formation in *C. jejuni* (**Figure 2A**). While iron is an essential nutrient, it may generate reactive oxygen species (ROS) through the Fenton/Haber–Weiss reaction (Cornelis et al., 2011). Since oxidative stress affects biofilm formation in *C. jejuni* (Oh and Jeon, 2014; Oh et al., 2016), we hypothesized that the iron-mediated biofilm promotion may be related to oxidative stress. To examine this hypothesis, we investigated the effect of antioxidant treatment on biofilm formation in the presence of iron. The levels of total ROS were increased by iron and reduced by an iron chelator and an antioxidant (**Figure 2B**). The intracellular levels of iron were increased by iron supplementation and reduced by an iron chelator (**Figure 2C**). Antioxidant treatment inhibited the iron-mediated promotion of biofilm formation, although the intracellular iron level of iron- and antioxidant-treated biofilms was comparable to that in the biofilms treated with only iron (**Figure 2C**). Interestingly, biofilm formation was enhanced in proportion to the level of total ROS (**Figures 2A,B**), not that of intracellular iron (**Figures 2A,C**). The viability of *C. jejuni* in biofilms was not affected by the treatment conditions used in the study (**Supplementary Figure S1B**). These results suggested that biofilm promotion by iron is associated with oxidative stress in *C. jejuni*.

### Increased Production of EPS by Iron

**FIGURE 3:**
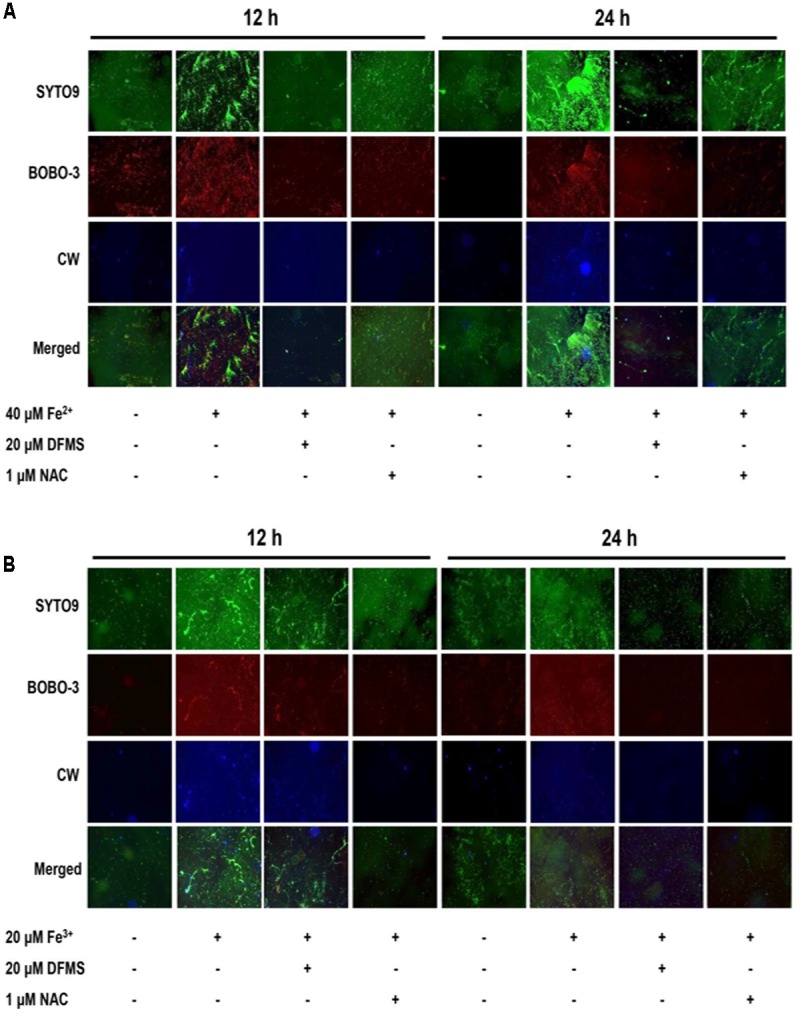
Stimulatory effects of Fe^2+^ **(A)** and Fe^3+^ **(B)** on biofilm formation in *C. jejuni* NCTC 11168. Biofilms were observed with fluorescence microscopy after staining with SYTO9, BOBO3, and calcofluor white (CW) to detect total DNA (i.e., biofilm structures), extracellular DNA, and extracellular polysaccharides, respectively. The experiments were repeated three times and generated similar results. DFMS and N-acetylcysteine (NAC) were used as an iron chelator and an antioxidant, respectively.

The formation of biofilms was observed in the presence and absence of iron using fluorescence microscopy. Iron supplementation significantly enhanced the establishment of microcolonies at the early stage (12 h) of biofilm formation and also increased the development of matured biofilm structures at 24 h (**Figure 3**), suggesting that iron may affect the early and late stages of biofilm formation in *C. jejuni*. To observe EPS production, biofilms were stained with BOBO3 and calcofluor white (CW) to detect eDNA and extracellular polysaccharides, respectively. BOBO-3 is a DNA-binding red fluorescent dye and cannot penetrate through the membrane and thus is used to detect eDNA. CW is a fluorescent dye that binds to β1–3 and β1–4 carbohydrate linkages and has been used to detect polysaccharides in *C. jejuni* biofilms (McLennan et al., 2008). Both Fe^2+^ and Fe^3+^ substantially increased the production of eDNA and extracellular polysaccharides in biofilms; however, an iron chelator and an antioxidant reduced the levels of eDNA and extracellular polysaccharides (**Figure 3**). These findings demonstrated that iron promoted biofilm formation in *C. jejuni* by stimulating the production of eDNA and extracellular polysaccharides.

### Effect of Iron on Biofilm Formation in 70 Strains of *C. jejuni* From Retail Raw Chicken

**FIGURE 4:**
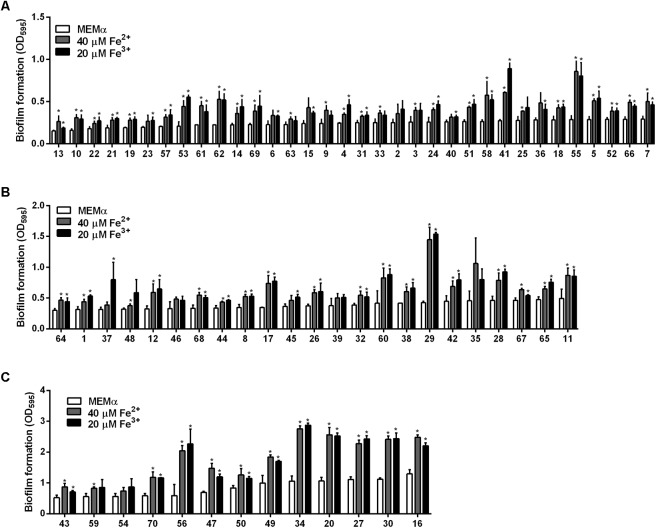
Effects of iron (40 μM Fe^2+^ and 20 μM Fe^3+^) on biofilm formation in 70 chicken isolates of *C. jejuni* that inherently form biofilms at low **(A)**, medium **(B)**, and high **(C)** levels. The inherent capability of biofilm formation was determined based on the OD_595_ in the biofilm assay. The low-, medium-, and high-level biofilm formers were those that generated OD_595_ of less than 0.3, between 0.3 and 0.5, and greater than 0.5, respectively, in the absence of iron. The results show the means and standard deviations of three samples in a single experiment. The experiments were repeated three times, and similar results were observed in all repeated experiments. The statistical analysis was performed with Student’s *t*-test compared to the control without iron treatment. ^∗^*P* < 0.05.

**FIGURE 5:**
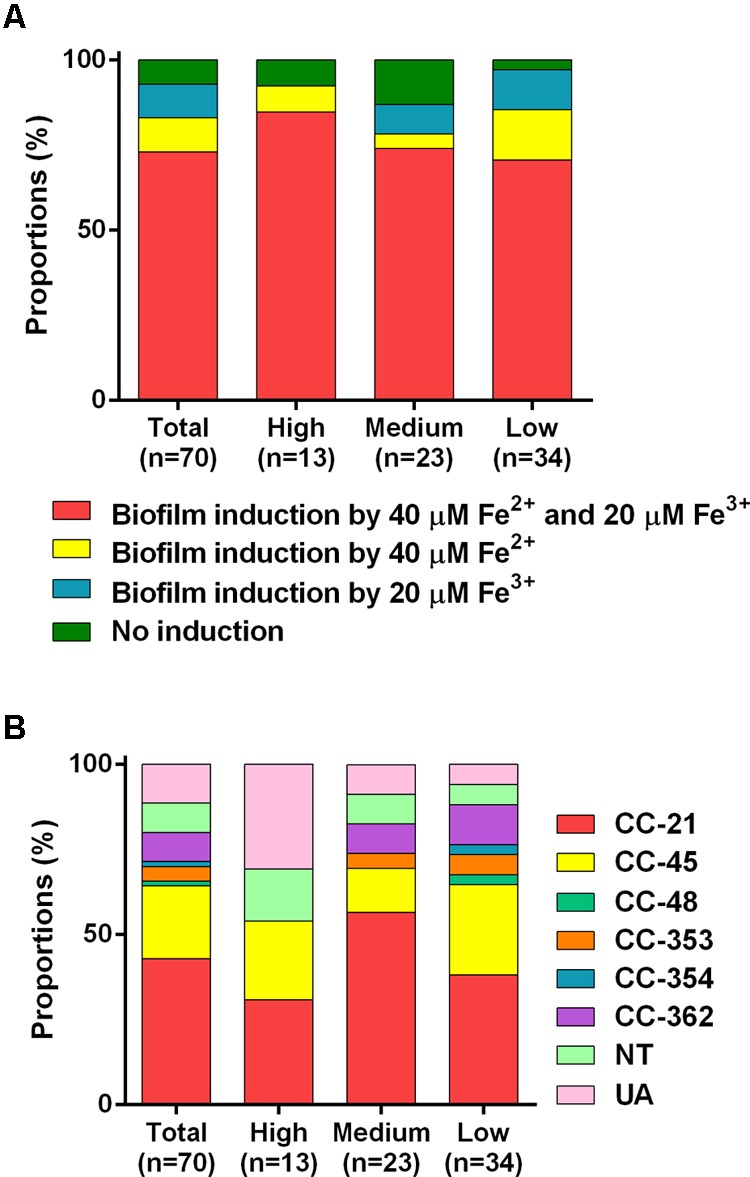
MLST clonal complexes (CCs) of 70 tested strains of *C. jejuni* with different biofilm formation capabilities **(A)**, and the proportions of the *C. jejuni* strains whose biofilm formation was enhanced by 40 μM Fe^2+^ and/or 20 μM Fe^3+^ **(B)**. The low-, medium-, and high-level biofilm formers are the strains that generated OD_595_ of less than 0.3, between 0.3 and 0.5, and greater than 0.5, respectively, in the absence of iron. The MLST CCs of the 70 *C. jejuni* strains tested in the study were determined in our previous report (Oh et al., 2015).

Using the iron concentrations determined with *C. jejuni* NCTC 11168 (40 μM Fe^2+^ and 20 μM Fe^3+^; **Figure 1**), the effect of iron on biofilm formation was evaluated in 70 *C. jejuni* strains that were isolated from retail raw chicken in our previous study (Oh et al., 2015). The levels of biofilm formation in the tested strains varied significantly in the absence of iron, ranging from low (**Figure 4A**), medium (**Figure 4B**), to high levels (**Figure 4C**), and iron significantly stimulated biofilm formation in the tested strains with strain-dependent variations (**Figure 4** and **Supplementary Figure S2**). Similar to *C. jejuni* NCTC 11168 (**Figure 1**), 51 (72.9%) of the 70 tested strains exhibited biofilm promotion by both 40 μM Fe^2+^ and 20 μM Fe^3+^ (**Figure 5A**). However, biofilm formation in 14 (20%) strains was enhanced by either only 40 μM Fe^2+^ or 20 μM Fe^3+^, not by both (**Figure 5A**), and biofilm formation in five strains (7.1%) was promoted by neither 40 μM Fe^2+^ nor 20 μM Fe^3+^ (**Figure 5A**). The intrinsic level of biofilm formation was not correlated to the multilocus sequence typing (MLST) clonal complexes (CCs) of the strains. Overall, MLST CCs 21 and 45 were distributed in weak-, medium-, and strong-biofilm formers; however, minor MLST CCs, such as 353, 354, and 362, were not found in strong-biofilm formers (**Figure 5B**).

**FIGURE 6:**
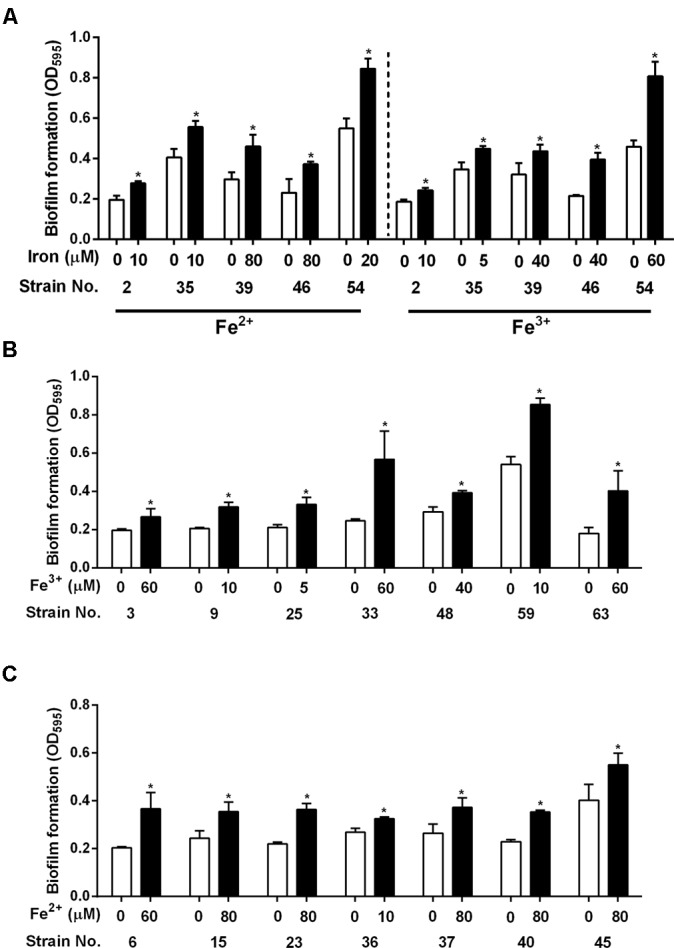
Biofilm stimulation at different iron concentrations in the *C. jejuni* strains whose biofilm formation was enhanced by neither 40 μM Fe^2+^ nor 20 μM Fe^3+^ **(A)**, by 40 μM Fe^2+^ but not by 20 μM Fe^3+^ **(B)**, and by 20 μM Fe^3+^ but not by 40 μM Fe^2+^ **(C)**. The results show the means and standard deviations of three samples in a single experiment. Similar results were observed in all experiments repeated three times independently. The statistical analysis was performed with Student’s *t*-test in comparison with the control without iron treatment. ^∗^*P* < 0.05.

Assuming strain variations in iron uptake and/or oxidative stress defense, we conducted biofilm assays at different iron concentrations with the 19 strains whose biofilm formation was not enhanced by either 40 μM Fe^2+^ or 20 μM Fe^3+^ or both (**Figures 4**, **5A**). Interestingly, biofilm formation in all tested strains was enhanced by both Fe^2+^ and Fe^3+^ at different concentrations with substantial strain variations (**Figure 6**). These results show that most *C. jejuni* strains increased biofilm formation in similar concentration ranges (ca. 40 μM Fe^2+^ and 20 μM Fe^3+^). However, different iron concentrations were required for biofilm stimulation in some *C. jejuni* strains.

## Discussion

As an essential nutrient, iron is involved in various biological processes in *C. jejuni* (Palyada et al., 2004). Fe^2+^ may diffuse through outer-membrane porins and then pass through FeoB in the cytoplasmic membrane in *C. jejuni* (Naikare et al., 2006; Miller et al., 2009). Fe^3+^ uptake is mediated by multiple membrane transporters, whose expression is regulated by the ferric uptake regulator Fur (Miller et al., 2009). The findings in this study demonstrate that both Fe^2+^ and Fe^3+^ stimulate biofilm formation in *C. jejuni*. Effects of iron on biofilm formation have also been reported in other bacteria. Iron-depleted saliva increases aggregation and biofilm formation of *S. mutans*, an important pathogen causing dental caries (Berlutti et al., 2004). Iron enhances biofilm formation in *S. aureus* ATCC 35556 and a few clinical strains of *S. aureus* (Lin et al., 2012). However, the effect of iron on biofilm formation appears to be strain-dependent in *S. aureus* since iron reduces biofilm formation in *S. aureus* Newman (Johnson et al., 2005). Biofilm formation in most of the strains tested in this study was stimulated 40 μM Fe^2+^ and 20 μM Fe^3+^ (**Figures 4**, **5A**), which were effective at biofilm promotion in *C. jejuni* NCTC 11168 (**Figure 1**). In some other *C. jejuni* strains, 40 μM Fe^2+^ and 20 μM Fe^3+^ did not enhance biofilm formation, and different iron concentrations were required to promote biofilm formation in these strains (**Figure 6**). Variations in the iron concentrations impacting biofilm formation may be associated with the strain variations in iron-associated genes in *C. jejuni*. Genes encoding iron transporters, which are found in the genome of some *C. jejuni* strains, have been found to be non-functional or absent in other strains (Miller et al., 2009). Presumably, the diversities in the genes involved in iron uptake might be associated with the strain-dependent variations in iron concentrations affecting biofilm stimulation. We could not observe any correlation between the MLST CCs and the levels of biofilm formation (**Figure 5B**), presumably because the MLST scheme is based on the polymorphisms of the seven housekeeping genes (*aspA, glnA, gltA, glyA, pgm, tkt*, and *uncA*) (Dingle et al., 2001). However, biofilm formation is complicated and involves proteins of various biological processes, such as motility, chemotaxis, oxidative stress response, heat shock response, and energy generation (Kalmokoff et al., 2006). This might be the reason why the MLST sequence types were not well correlated with biofilm formation in this study.

Biofilm formation in *P. aeruginosa* is inhibited by lactoferrin, a mammalian iron chelator (Singh et al., 2002). The sequestration of iron by lactoferrin reduces the intracellular levels of iron and inhibits biofilm formation (Banin et al., 2005). The anti-biofilm effect of lactoferrin in *P. aeruginosa* involves incessant twitching motility that affects bacterial attachment to a surface and microcolony formation during biofilm development (Singh et al., 2002). Twitching motility is mediated by type IV pili (Mattick, 2002). Similarly, iron stimulates biofilm formation in *E. coli* by controlling the expression of type I fimbriae (Wu and Outten, 2009). However, *C. jejuni* does not produce pili (Gaynor et al., 2001). This indicates that a different mechanism may be involved in iron-mediated biofilm promotion in *C. jejuni*.

It has been reported that oxidative stress affects biofilm formation in *C. jejuni* (Kim et al., 2015). A mutation of *ahpC* leads to the accumulation of total ROS and lipid hydroperoxides and enhances biofilm formation in *C. jejuni*, and antioxidant treatment inhibits the biofilm promotion by an *ahpC* mutation (Oh and Jeon, 2014). PerR and CosR, key oxidative stress defense regulators in *C. jejuni*, also consistently affect biofilm formation in *C. jejuni* (Oh and Jeon, 2014; Turonova et al., 2015). Although *C. jejuni* is microaerophilic, interestingly, aerobic exposure facilitates biofilm formation in *C. jejuni* (Reuter et al., 2010). Our previous study has shown that oxidative stress plays a role in biofilm stimulation in *C. jejuni* under aerobic conditions (Oh et al., 2016). Oxidative stress affects biofilm formation in some other bacteria. Biofilm formation in *Mycobacterium avium* increases by the autoinducer-2 (AI-2) signaling molecules by the induction of oxidative stress response involving the upregulation of genes encoding alkyl hydroperoxidases (e.g., *ahpC* and *ahpD*), not through quorum sensing (Geier et al., 2008). An *ahpC* mutation in *Acinetobacter oleivorans* DR1 increases the accumulation of H_2_O_2_ in the cell, which enhances biofilm formation by the induction of exopolysaccharide production in biofilms (Jang et al., 2016). In *C. jejuni*, iron supplementation increased the accumulation of total ROS (**Figure 2B**) and the production of eDNA and extracellular polysaccharides in *C. jejuni* biofilms (**Figure 3**). Aerobic exposure and iron supplementation commonly result in the increase in oxidative stress. Based on the findings of this study, biofilm stimulation by iron through oxidative stress in *C. jejuni* may involve EPS production (**Figure 3**). EPS constitutes over 90% of the dry mass of biofilms (Flemming and Wingender, 2010) and contributes to the nutrient acquisition and desiccation tolerance (Flemming et al., 2016). Similarly, biofilm stimulation by iron in *S. aureus* is mediated by the increased production of polysaccharide intercellular adhesin (i.e., β-1,6-linked N-acetyl glucosamine polymer) involved in biofilm formation (Lin et al., 2012). Exposure to increased iron concentrations augments the accumulation of ROS in *C. jejuni* (**Figure 2B**) and the production of EPS in biofilms (**Figure 3**). Presumably, the enhanced production of EPS by iron may help *C. jejuni* to reduce exposure to oxygen and other stress conditions by facilitating the formation of biofilm matrices encasing *C. jejuni*.

Cationic metal ions can be toxic for planktonic bacterial cells at high concentrations; however, the absorption and accumulation of metal ions in biofilms stabilizes biofilms and prevents their erosion by shear forces in *B. subtilis* (Grumbein et al., 2014). EDTA disrupts *P. aeruginosa* biofilms and enhances the dispersal of bacterial cells from biofilms (Banin et al., 2006). However, Mg^2+^, Ca^2+^, and Fe^2+^ inhibit the effect of EDTA on biofilm disruption, suggesting that divalent cations are important components that stabilize biofilms in *P. aeruginosa* (Banin et al., 2006). In addition to the effect of iron on oxidative stress, we cannot exclude the possibility that iron may also be involved in the stabilization of biofilm structure in *C. jejuni*. Future studies are required to elucidate the molecular mechanisms underlying the interplay between iron and oxidative stress in biofilm formation in *C. jejuni*.

## Materials and Methods

### Bacterial Strains and Culture Conditions

*Campylobacter jejuni* NCTC 11168, the first genome-sequenced strain, was primarily used in this study. Seventy strains of *C. jejuni* were isolated from raw chicken in our previous study (Oh et al., 2015). The *C. jejuni* strains were routinely maintained at 37°C under microaerobic conditions (5% O_2_, 10% CO_2_, and 85% N_2_) on either Mueller-Hinton agar plates or MEMα (Gibco, #41061-029), which does not contain iron. The microaerobic conditions were generated using a cylinder containing the premixed gas.

### Biofilm Assays

Biofilm assays were carried out according to a protocol described in our previous study using MEMα (Oh and Jeon, 2014). Briefly, bacterial suspension was prepared from an overnight culture and then diluted with fresh MEMα to an OD_600_ of 0.07 and placed into a 96-well plate (Corning, #3595) in the presence of iron (Fe^2+^ or Fe^3+^), iron chelator (Deferoxamine mesylate, DFMS), or antioxidant (N-acetyl-L-cysteine, NAC). After 24 h, biofilms were washed twice with PBS (pH 7.4) and stained with 1% crystal violet. The dye eluted with the elution buffer (10% acetic acid and 30% methanol) was measured with a plate reader (FLUOstar Omega; BMG Labtech, Germany) at 595 nm. For bacterial counting of biofilms, *C. jejuni* biofilm samples were washed twice with PBS and resuspended in fresh MH broth. The resuspended biofilm samples were serially diluted in MH broth and spread on MH agar for enumeration. The experiment was conducted with triplicate samples and independently repeated at least three times.

### Measurement of Total ROS

The total ROS level in biofilms was determined with CM-H_2_DCFDA (Thermo Fisher, United States), a general oxidative stress indicator, according to our previous study (Oh and Jeon, 2014). Briefly, biofilms were washed twice with PBS and re-suspended in PBS (pH 7.4). After addition of 10 μM CM-H_2_DCFDA, fluorescence was measured with a fluorometer (FLUOstar Omega) at ex 485 nm/em 520 nm. The total ROS levels were normalized with the total protein amounts that were determined using a Bradford assay. The experiment was conducted with triplicate samples and independently repeated three times.

### Fluorescence Microscopic Analysis of Biofilms

Biofilm formation was also analyzed with fluorescence microscopy. Biofilms were developed on a circle cover glass in a 24-well plate for 24 h at 37°C under microaerobic conditions. Biofilm samples were washed twice with PBS and fixed with 4% paraformaldehyde for 30 min at room temperature. The biofilms were then washed with PBS and stained with SYTO9, BOBO3, and CW to detect total (both intracellular and extracellular) DNA, intracellular DNA, and extracellular polysaccharides, respectively. CW binds β1–3 and β1–4 carbohydrate linkages and was previously used to detect extracellular polysaccharides in biofilms (McLennan et al., 2008). After washing, the biofilms were analyzed with a fluorescence microscope (Carl Zeiss, Axio Imager A1). The experiment was repeated three times.

### Measurement of eDNA in Biofilms

The isolation of eDNA from biofilms was performed as described previously (Wu and Xi, 2009). After washing twice with PBS, biofilms were harvested with 2% EDTA and incubated at 4°C for 3 h with shaking (250 rpm). An equal volume of 2% cetyltrimethyl ammonium bromide (CTAB) was added, and the suspension was incubated on ice for 1 h. After centrifugation at 10,000 ×*g* for 10 min, the pellet was re-suspended in TE buffer, and an equal volume of phenol: chloroform: isoamyl alcohol (25: 24: 1) solution was added. After centrifugation, the top phase of each sample was transferred to a new tube, and 2× volume of ice-cold ethanol and 1/10× volume of 3 M sodium acetate were added. After incubation at -20°C for 1 h, the pellets were washed twice with 70% ethanol. After dissolving with water, DNA concentrations were measured with a spectrophotometer and normalized with the amount of total proteins in biofilms that was determined with a Bradford assay. The experiment was conducted with triplicate samples and repeated three times.

### Measurement of Intracellular Iron Levels

The intracellular iron concentration was measured as described previously with slight modifications (Riemer et al., 2004). Briefly, biofilms were washed twice with PBS and disrupted with a sonicator (BioRuptor Plus; Diagenode, United States). The biofilm samples were mixed with an iron-detection reagent (6.5 mM ferrozine, 6.5 mM neocuproine, 2.5 M ammonium acetate, and 1 M ascorbic acid) and incubated at room temperature for 30 min. The absorbance at 550 nm was measured with a plate reader (FLUOstar Omega). Intracellular iron levels were normalized with protein concentrations that were determined with a Bradford assay. The experiment was conducted with triplicate samples and independently repeated three times.

### Statistical Analysis

The statistical analysis was performed with Student’s *t*-test in comparison with the control without iron treatment using GraphPad Prism 6 (GraphPad Software, La Jolla, CA, United States).

## Author Contributions

EO and BJ designed the project. EO and KA performed the experiments. EO and BJ data analysis. EO, KA, and BJ wrote the manuscript.

## Conflict of Interest Statement

The authors declare that the research was conducted in the absence of any commercial or financial relationships that could be construed as a potential conflict of interest. The reviewer GP and handling Editor declared their shared affiliation.
