# Supplementary material for: Enhanced Biofilm Formation by Ferrous and Ferric Iron Through Oxidative Stress in Campylobacter jejuni
Source: Front Microbiol. 2018 Jun 6;9:1204. doi: 10.3389/fmicb.2018.01204 (PMC5998592; doi:10.3389/fmicb.2018.01204)
Supplement: Supplementary file 1 [file Presentation_1.pdf]

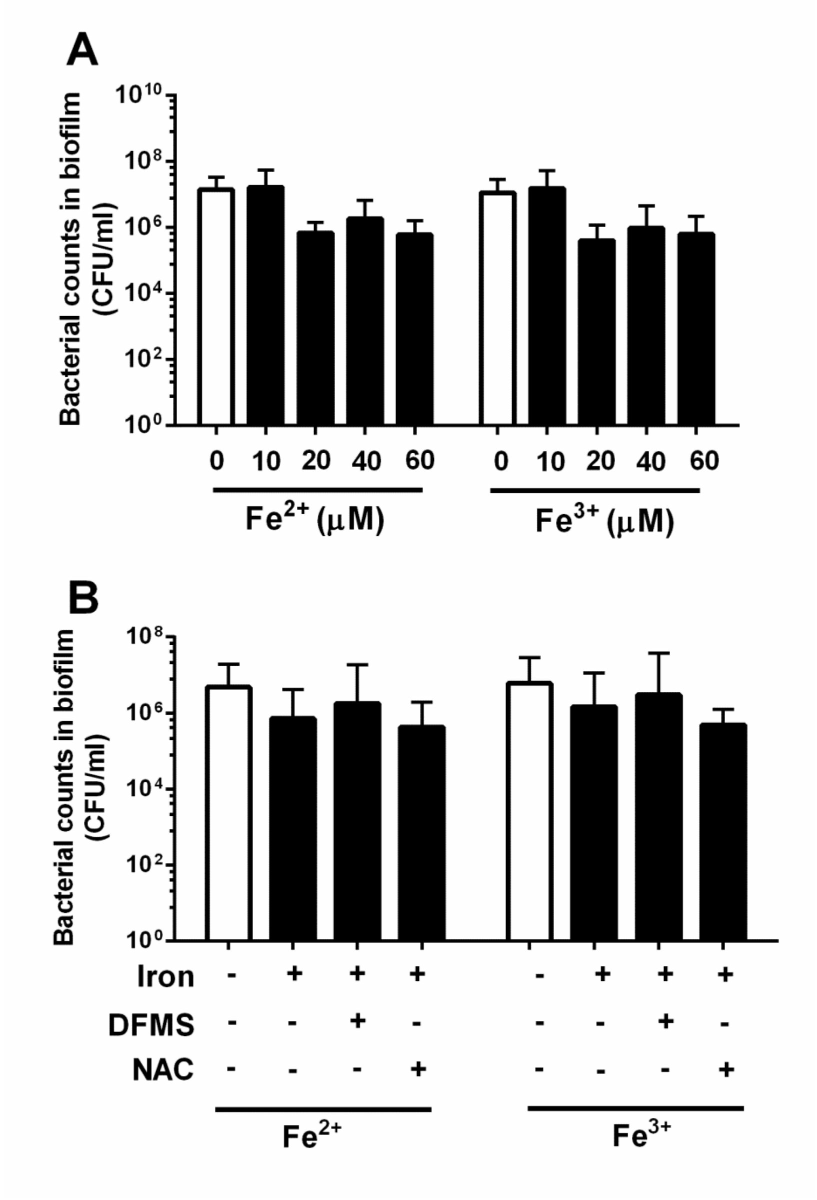

Fig. S1. ***C. jejuni*** **counts in biofilms at different iron concentrations (A) and under the treatment conditions of iron (40 µM Fe_{2+} and 20 µM Fe_{3+}), 20 µM DFMS (deferoxamine mesylate, an iron chelator), and 1 µM NAC (N-acetylcysteine, an antioxidant) (B)**. Biofilms were incubated under microaerobic conditions for 24 h before enumeration. The experiment was conducted with triplicate samples and independently repeated three times. *C. jejuni* NCTC 11168 was used in the experiment.

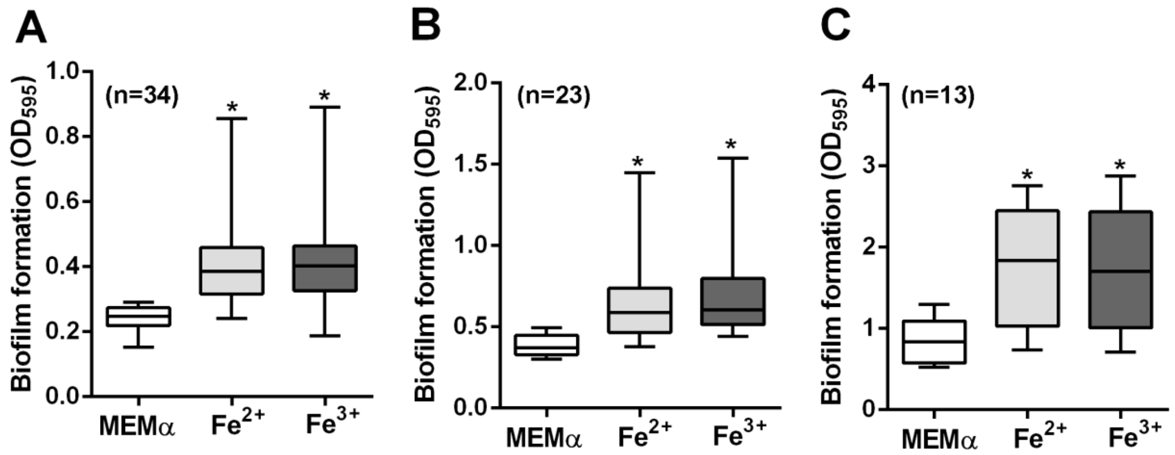

Fig. S2. Increased biofilm formation by iron in 70 chicken isolates of *C. jejuni* forming biofilms at low (A), medium (B), and high (B) levels. Biofilms were treated with 40 μM Fe^{2+} and 20 μM Fe^{3+}. The experiment was carried out with triplicate samples and repeated three times. The statistical analysis was performed with student's *t*-test compared to the untreated samples (MEMα). *: $P<0.05$.
